# Incidence of End Stage Renal Disease among Type 1 Diabetes: A Nationwide Cohort Study in Taiwan

**DOI:** 10.1097/MD.0000000000000274

**Published:** 2014-12-02

**Authors:** Wei-Hung Lin, Chung-Yi Li, Wei-Ming Wang, Deng-Chi Yang, Te-Hui Kuo, Ming-Cheng Wang

**Affiliations:** From the Institute of Clinical Medicine, College of Medicine, National Cheng Kung University (WHL), Division of Nephrology, Department of Internal Medicine, National Cheng Kung University Hospital, College of Medicine, National Cheng Kung University (WHL, DCY, THK, MCW), Department and Graduate Institute of Public Health, College of Medicine, National Cheng Kung University (CYL, THK), Biostatistics Consulting Center, National Cheng Kung University Hospital, College of Medicine (WMW), Institute of Gerontology, College of Medicine, National Cheng Kung University (DCY), Institute of Clinical Pharmacy and Pharmaceutical Sciences, College of Medicine, National Cheng Kung University (MCW) Tainan; and Department of Public Health, College of Public Health, China Medical University (CYL) Taichung, Taiwan.

## Abstract

The aim of our study was to estimate the risk of end-stage renal disease (ESRD) in type 1 diabetes mellitus (T1DM) in Taiwanese people; in addition, our goal was to determine how the age, year, and sex at registration of T1DM affects the risk.

Population-based cohort study.

A nationwide cohort study of 7203 Taiwanese patients with T1DM registered in 1999 to 2010 was followed up until ESRD, death, or the end of follow-up on December 31, 2010.

Annual age-, sex-, and calendar year-specific incidence rates of ESRD of the general population were used to calculate the standardized incidence ratio (SIR) of ESRD in relation to T1DM.

The SIR of ESRD for male and female patients with T1DM was significantly increased at 25.85 (95% CI 23.40–28.29) and 28.08 (95% 25.45–30.71), respectively; the peak was at age 15 to 29 years for both genders. The cumulative incidence of ESRD was similar in male and female patients but was significantly higher in patients ≥ 30 years old than in patients <30 years old (10.25% vs. 3.57%, *P* < 0.001). Patients aged <15 years had a significantly lower risk of ESRD as compared to those aged 15 to 29 years; patients aged 30 to 44 (adjusted HR, 1.491) and 45 to 60 years (adjusted HR, 2.111) showed significantly increased hazards. Our data also demonstrated a lower risk of ESRD in patients who were registered in later years than in earlier years.

The risk of ESRD is substantially increased in T1DM in the ethnic Chinese population. The continuously declining risk of ESRD in T1DM may advocate the use of a multidisciplinary chronic kidney disease care system in Taiwan.

## Abstract 2

Supplemental Digital Content is available in the text

## INTRODUCTION

Type 1 diabetes mellitus (T1DM) is identified as being associated with a higher age-adjusted mortality risk of death when compared to the general population.^1,2^ T1DM can lead to hyperglycemia, which is linked to several acute (eg, diabetic ketoacidosis) and chronic (eg, diabetic nephropathy and cardiovascular disease) complications.^3^ T1DM has been recognized as an important etiology of diabetic nephropathy, which has been recognized as one of the most common complications; it is also a major predictor of premature death.^4^ Ethnic differences are likely to cause different incidence rates of diabetes and accompanying vascular complications.^5,6^ It is well known that the incidence of T1DM is increased among Caucasoid populations compared to Mongoloids, Blacks, and Chinese; such ethnic diversity tends to show the importance of differential genetic susceptibility among different populations.^7^

The number of new cases of T1DM in European children less than 5 years is predicted to double between 2005 and 2020; in addition, the prevalence of cases in children under 15 years old will increase by 70%.^8^ Our previous study also found a significant increasing trend for T1DM in males and females aged <15 years.^7^ Moreover, Taiwan has been a country with a very high prevalence of end stage renal disease (ESRD), with diabetes believed to be the leading cause.^9^ Particularly, subjects with a low socio-economic and educational status have a lower awareness of chronic kidney disease (CKD)^10^; this reiterates the importance of early diagnosis. Although the cumulative incidence of ESRD has ranged from 4% to 17% at 20 years after T1DM diagnosis^4,11,12^ (noted in different populations), very few studies regarding the ethnic Chinese population have been described. It is well-known that African-American and Asian (Taiwanese and Japanese) people have a greater risk for developing ESRD, when compared to Caucasians, in all cohorts.^9,13^ Asians constitute a large portion of non-Black groups in the U.S. and other developed countries, but there is limited data focusing on ESRD from T1DM in Asian populations.

Using a large population-based cohort with T1DM in Taiwan, registered between 1999 and 2010, we reported the long-term mortality trends in patients of all ages with T1DM.^7^ We have further studied the long-term risk of ESRD in patients with T1DM and examined how the age at registration of diabetes, time of registration, and sex affect these risks.

## RESEARCH DESIGN AND METHODS

### Data Sources

The National Health Insurance (NHI) program was launched in Taiwan on March 1, 1995. The National Health Insurance Research Database (NHIRD), a large scale computerized database supervised by Bureau of NHI, Department of Health, and maintained by the National Health Research Institutes, is provided to local scientists in Taiwan for research purposes. The NHIRD includes all inpatient and ambulatory medical claims for approximately 99% of Taiwanese people; information obtained from the NHIRD is considered to be complete and accurate.^14^ Data of NHIRD which can be used to identify patients or care providers, including medical institutions and physicians, are scrambled before it is sent to the National Health Research Institutes for database construction. Data are further scrambled before it is released to each researcher. Therefore, individual patient or health care providers cannot be identified from the database.

In Taiwan, the certification of various catastrophic illnesses is subject to evaluation and review by the Bureau of NHI. Patients with catastrophic illness certificates (CICs) are eligible for exemption from insurance premiums and co-payments. Therefore, the CICs data are accurate and reliable.^15^ The CICs will be terminated once the patient's die. Access to the NHIRD by this study has been approved by the Review Committee of the NHRI; this study was approved by the Institutional Review Board of National Cheng Kung University Hospital.

### Identification of Newly registered T1DM and ESRD Patients

In this nationwide cohort study, we searched the Taiwan NHIRD for the T1DM population from January 1, 1999 to December 31, 2010 (as per the International Classification of Diseases, 9th Revision, Clinical Modification, ICD-9-CM code 250). We considered the first registered CICs for T1DM and ESRD as the patients with newly registered T1DM or newly diagnosed ESRD, respectively; we excluded all CICs registered before 1999. T1DM and ESRD are recognized as catastrophic illnesses by the Taiwan NHI. Because no co-payment is required for admission, emergency, and outpatient services, this certification is only applied when detailed and specific clinical data are met. The CICs registration is a valid and reliable source of data for the T1DM and ESRD retrieval.^15^ The term “diagnosed” in our study was defined as the disease or condition which causes a person's signs and symptoms, as determined by a medical examination. The term “registered” in our study was defined as patients diagnosed with T1DM or ESRD, which applied for catastrophic illness registration cards from the Bureau of NHI; these patients do not need to make co-payments when seeking health care for catastrophic illnesses. The patients with ESRD that need regular dialysis normally apply for CIC shortly after they are newly diagnosed with ESRD; this is due to the enormous associated costs in our medical system. Patients with T1DM, on the other hand, may have a time gap between “diagnosis” and “registration” due to minor conditions or the slow progression of T1DM.

We also validated the ICD-9-CM codes used in the identification of T1DM by analyzing the chart records, as previously described.^7^ In short, T1DM was classified in patients who had three or more outpatient diabetes diagnoses with insulin prescriptions and either a history of diabetic ketoacidosis, a positive glucagon test, or the presence of glutamic-acid-decarboxylase antibodies.^16^ Among the randomly selected 60 patients coded with T1DM in the National Cheng Kung University Hospital, 59 were confirmed by chart review, yielding a positive predictive value of 98.3%.

### Statistical Analysis

Patients were followed from registration of T1DM to the occurrence of ESRD, death, or end of follow-up on December 31, 2010. We first calculated bi-annual incidence rates of ESRD (cases per 1000 inhabitants) in patients with T1DM, according to sex and age (<15, 15–29, 30–44, 45–59, and ≥60 years). The bi-annual incident rate of ESRD among T1DM was calculated by dividing the number of incident ESRD cases by the averaged mid-year population of T1DM for every 2 years. To examine the secular trend of ESRD incidence rates within the study period, we treated the calendar year as a continuous variable; we tested the statistical significance of the regression coefficient derived from the multivariable Poisson regression model, which simultaneously included age, sex, and calendar year. We also use the 2000 WHO standard population as the reference in order to calculate the age- and sex-standardized overall ESRD bi-annual incidence rate.

To estimate the relative risk, indicated by the age, sex, and calendar year (1999–2010) standardized incidence ratios (SIR) of ESRD in patients with T1DM, we compared the risk of ESRD in T1DM with that of Taiwan's general population; for the general population, we used the same age and sex distributions retrieved from Department of Statistics, Ministry of the Interior, Executive Yuan, Taiwan. The 95% confidence intervals (CIs) of SIR were estimated using the Poisson distribution. Kaplan–Meier estimates of ESRD were plotted and differences between the two groups were examined by the log-rank test. Cumulative incidence of ESRD was also calculated by the Kaplan–Meier method. Patients were censored on December 31, 2010.

We performed multiple proportional sub-distribution hazards regression models; these models considered death as a competing risk event in order to estimate the adjusted hazard ratios (HRs) of ESRD associated with sex, age at registration, and time period of registration. The sub-HR of competing risk regression was computed using the “stcrreg” command in STATA 12.0 (StataCorp LP, College Station, TX), which is based on Fine and Gray proportional subhazards model.^17^ Data analysis was conducted using professional statistical packages, SPSS for Windows, version 17.0 (SPSS Inc, Chicago, IL), and STATA for Windows, version 12.0. A two-tailed *P* value below 0.05 was considered statistically significant.

## RESULTS

### Incidence of ESRD Among Patients With T1DM

Table 1 shows the bi-annual overall, as well as age- and sex-specific, incidence rates of ESRD among registered T1DM from 1999 to 2010. The standardized bi-annual overall incidence rate increased from 1999–2000 (0.13 per 1000) to 2003–2004 (3.13 per 1000), with a small declined thereafter; it again increased from 2009 to 2010 (3.52 per 1000). Throughout the study period, the β value was 0.08, with a significant secular trend (*P* < 0.001). Regardless of gender, and increased age-specific ESRD incidence rate was noted in the older groups (≥30 years); the highest incidence rate was found in those from 45 to 59 years old. Additionally, the incidence rate was zero in female subjects aged <15 years during 1999 to 2010. The most notable increase in age specific incidence rates for male and female subjects was 30 to 44 years (β = 0.199) and 15 to 29 years (β = 0.162), respectively. (See Table 1, Supplemental Content http://links.lww.com/MD/A94, which illustrates the annual sex- and age-specific prevalent case number of type 1 diabetes in Taiwan, 1999–2010.) (See Table 2, Supplemental Content http://links.lww.com/MD/A94, which illustrates annual sex- and age-specific incident case of ESRD among type 1 diabetes in Taiwan, 1999–2010.)

**TABLE 1:**
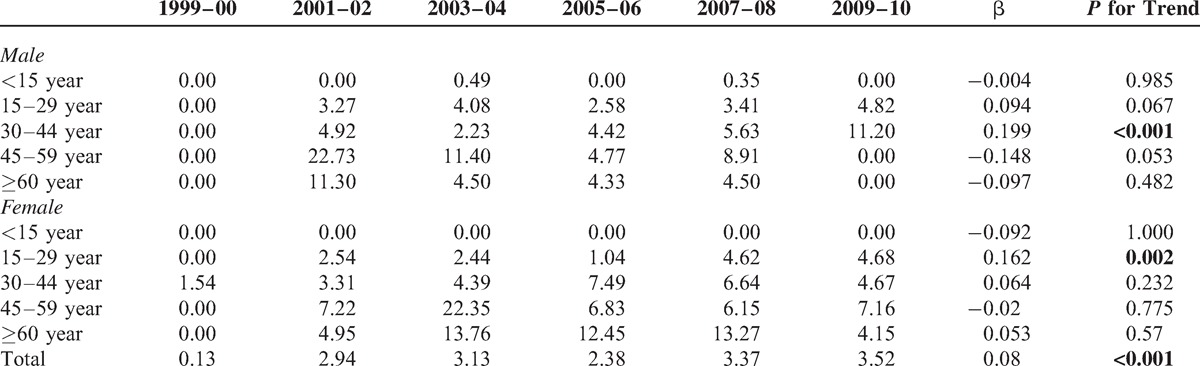
Bi-Annual Sex- and Age-Specific Incidence Rate (Per 1000 Inhabitants) of End Stage Renal Disease Among Patients With Type 1 Diabetes in Taiwan, 1999 to 2010

### SIR of ESRD Among Patients With T1DM

The sex-specific ESRD SIR for male and female patients with registered T1DM was significantly increased at 25.85 (95% CI 23.40–28.29) and 28.08 (95% CI 25.45–30.71), respectively (Table 2). Among the age stratifications, the SIR peaked for patients whose age at registration was 15 to 29 years (males: 167.79 [95% CI 142.78–192.80]; females: 170.60 [95% CI 146.71–194.49]). In fact, the SIRs were significantly increased at all age stratifications for both genders; the SIR tended to decrease with advancing age, except male patients aged <15 years.

**TABLE 2:**
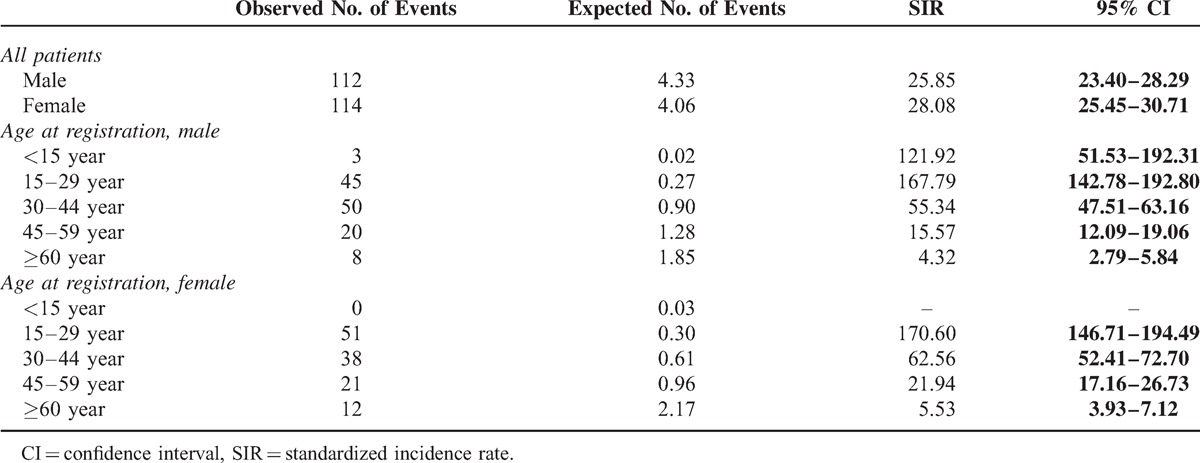
Standardized Incidence Ratio of End Stage Renal Disease Among Patients With Type 1 Diabetes in Taiwan According to Sex and Age at Registration, 1999–2010

### Adjusted Hazard Ratios and Cumulative Incidence of ESRD after Adjustment for Competing Risk of Death Among Patients With T1DM

**FIGURE 1:**
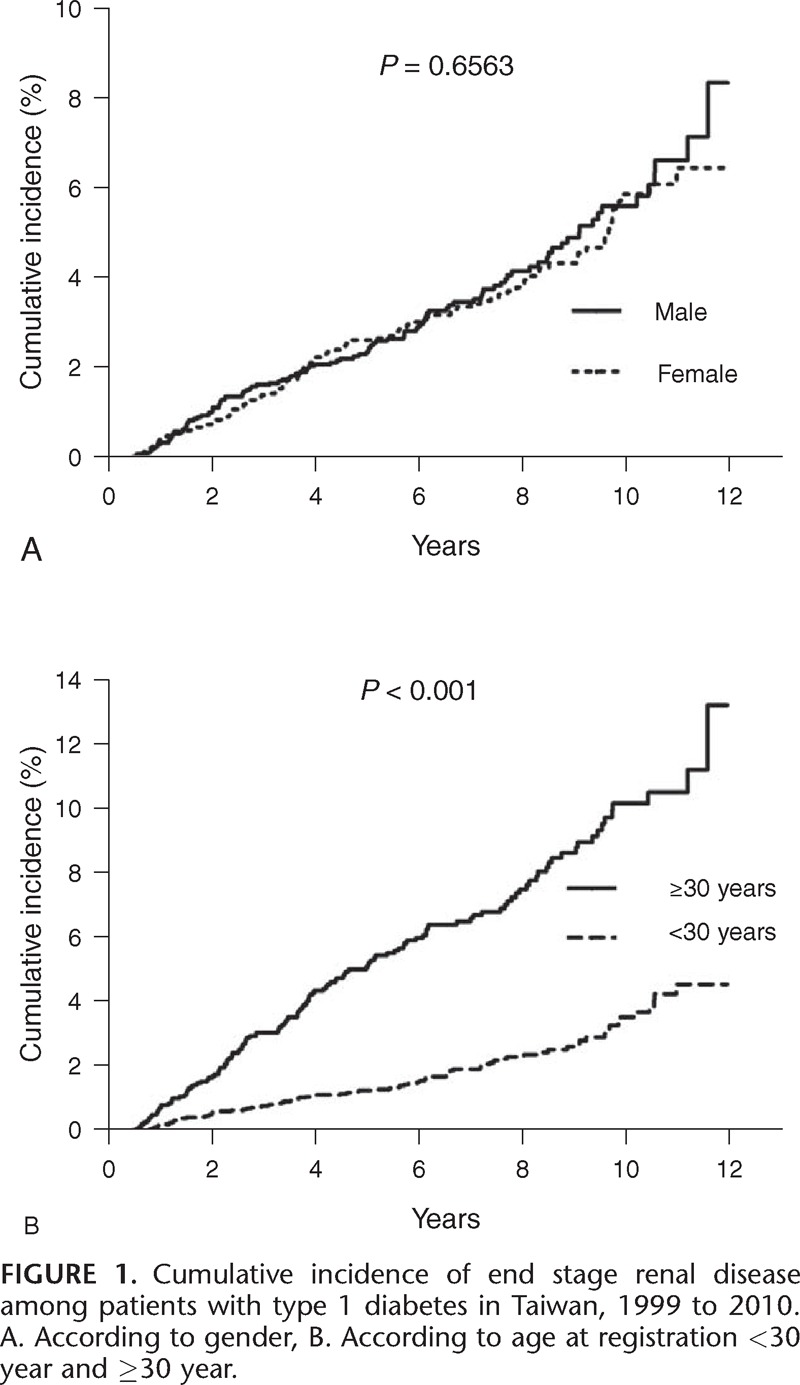
Cumulative incidence of end stage renal disease among patients with type 1 diabetes in Taiwan, 1999 to 2010. A. According to gender, B. According to age at registration <30 year and ≥30 year.

The 10-year cumulative incidence of ESRD among patients with registered T1DM in Taiwan was 5.62% in male and 5.88% in females, but this difference was statistically insignificant (*P* = 0.6563) (Figure 1A). However, the 10-year cumulative incidence was significantly higher in age at registration ≥30 years when compared with <30 years (10.25% vs. 3.57%, *P* < 0.0001) (Figure 1B). The multivariate analysis suggested that age at registration and time period of registration were significantly associated with the risk of developing ESRD; there was no significant association with sex (Table 3.) Patients aged <15 years had a significantly lower risk than patients in the other age groups; the risk of ESRD increased with age up to 60 years old. Compared to those with T1DM registered in 1999 to 2002, patients with T1DM who registered in 2003 to 2006 (adjusted HR, 0.646) and 2007 to 2010 (adjusted HR, 0.430) experienced significantly reduced risk of ESRD, suggesting a continuous improvement in prognosis of patients with T1DM in Taiwan.

**TABLE 3:**
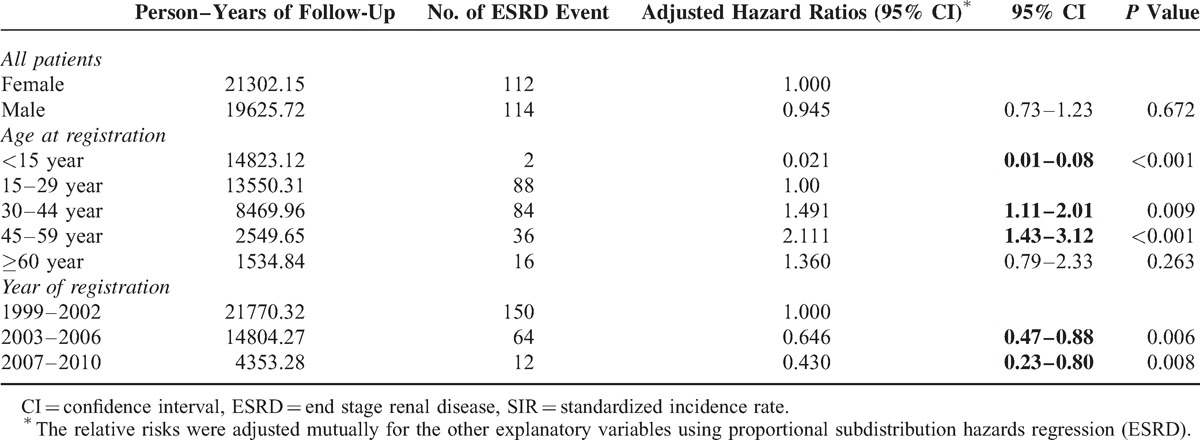
Hazard Ratios of End Stage Renal Disease Associated With Sex, Age, and Year of Registration in Patients With Type 1 Diabetes, 1999 to 2010, in Competing Risk Analysis

## DISCUSSION

Our study is the largest population-based T1DM cohort study focusing on ESRD incidence, with a follow-up period of up to 12 years in the ethnic Chinese population. We noticed that the incidence rates of ESRD among patients with T1DM increased significantly from 1999 to 2010, with higher age-specific ESRD incidence rates noted in the age at registration ≥30 years group; the highest incidence rate was from 45 to 59 years old. The SIRs of ESRD for male and female patients were both significantly increased; they peaked in those with age at registration of 15 to 29 years in both genders. The cumulative incidence of ESRD among patients with T1DM in Taiwan was similar in both genders, but was significantly higher in patients registered at ≥30 years when compared to those aged <30 years. We also noted that both age at registration and time period of registration were associated with the risk of developing ESRD; this suggested that there was continuously improved care of T1DM in Taiwan.

Our study included more than 7000 patients with T1DM; of this population, 226 patients developed ESRD. To date, this is the largest study estimating the risk of ESRD in Asian societies. A Finnish population-based study is the largest trial to estimate cumulative risk of ESRD in patients with T1DM. In Finland, T1DM accounted for one third of diabetic ESRD cases.^18^ The Finnish study found that the cumulative incidence among patients with T1DM for development of ESRD within 30 years was approximately 7.8%; in patients whose diagnosis of diabetes occurred before age 5 years, the risk was found to be significantly lower. The risk of ESRD was also lower for patients whose diagnosis occurred in more recent years.^19^ Notably, the study population was derived from a country with a fairly homogeneous health care system; almost all patients were white. Using different ethnic groups and health care systems, our study derived similar conclusions; patients with registered age under 15 years had a lower risk for ESRD and there was a continuously improved renal outcome over time. The decreased incidence of ESRD may have resulted from the success of an advanced CKD care system under nephrologists-based multidisciplinary care.^9,20^ The worldwide trend of an increased age of onset of ESRD cases caused by T1DM has been shown in the Joslin cohort.^21^ They found that the majority of ESRD occurred between ages 36 and 52 years, with the average duration of diabetes ranging from 21 to 37 years. The U.S. Renal Data System (USRDS) (http://www.usrds.org/) also showed that the number of ESRD cases considerably increased (almost doubled) in a patient aged 40 to 49 years with T1DM between 1990 and 2006 in the white U.S. population, but decreased in those 20 to 39 years old between. The increase in age at ESRD onset suggests an improvement in glycemic control that may delay the onset of macroalbuminuria and the subsequent onset of ESRD. This may explain our findings that the higher age-specific ESRD incidence rate was found in the middle aged groups (>30 years) and was highest from 45 to 59 years. Our findings, based the ethnic Chinese population, were similar to the results from both the Joslin cohort and the USRDS data.

Based on earlier reports, male sex has been considered as a traditional risk factor for ESRD incidence in T1DM.^4,5^ The development of kidney disease in patients with T1DM is thought to be more prevalent in men. There has been an increased incidence in cases of ESRD in male patients with T1DM in recent studies.^19,22^ The increased risk of ESRD in men noted in the earlier cohort was possibly due to a discrepancy in HbA1c levels, hypertension, and lipid levels between males and females. The improved management of these risk factors might explain the similar incidence rates between males and females in cases of kidney disease in recent cohort studies which were conducted in Western societies.^22^ Our study showed similar hazard ratios of ESRD for both genders in patients with T1DM, but showed a slightly increase SIR in female patients.

Recently, many studies have focused on the ethnic differences in both the rates of progression and outcomes of ESRD. Ethnic differences in CKD progression are not well characterized, but are of particular interest within most countries. Most of the literature has compared African-American to Caucasian outcomes in the U.S.; these studies have found that African-American have a significantly higher incidence of ESRD, suggesting a faster rate of progression through CKD.^23,24^ In addition, the developmental rates of African-American ESRD resulting from T2DM far exceeds those of Caucasians.^25^ Compared to Caucasians, American Asians had a higher incidence of ESRD^26^ and had a faster progression of CKD.^27^ Among diabetic patients, Asians had a significantly higher incidence of ESRD than Caucasians. Although the type of diabetes was not specifically mentioned or defined in these studies, most patients present with T2DM.^28^ In a cohort study, patients from South-Asian with T2DM had a higher incidence of microalbuminuria and faster progression of renal failure than Dutch-European patients. This discrepancy could be explained by either a higher incidence of nephropathy in the Asian diabetic patients and/or faster progression to ESRD.^29^ The racial difference (excess incidence of ESRD treatment among non-White North Americans) is particularly striking in presumed nephrosclerosis from T2DM; however, the association between ESRD and T1DM remains to be identified. There may be some disparities between ethnic groups and genetics, social, and environmental factors (such as diet, exercise, alcohol, smoking, and other exposures).^27^ It is difficult to understand the impact of these environmental factors on the outcome due to the lack of detailed information in our database; we believe our study will help to identify ethnic variables responsible for in type 1 diabetic ESRD.

Because death occurring before ESRD is a potential competing risk factor, the reduction in the risk of ESRD in patients with T1DM (especially in older patients) could have contributed to the number of earlier deaths prior to the development of ESRD; these factors included various causes of premature mortality, such as cardiovascular disease or complications of diabetes. Individuals with poor renal function are more likely to develop ESRD than to die, whereas those with more preserved renal function were more likely to die than to reach ESRD.^30^ In this study, we performed a competing risk regression analysis to account for the above-mentioned potential bias. Our data shows relative risk estimates of ESRD associated with sex, age, and year of registration in T1DM, based on the nationwide database. These data may be used for advising individual patients regarding goals and strategies for preventing premature mortality and ESRD.

Our study demonstrated that the incidence rate of ESRD peaked at ages of 45 to 59, but declined after 60 years. A recent population-based study found a higher risk of incidence of ESRD in the first 15 years following T1DM onset. The covariate adjusted HR of ESRD was 1.31 (95% CI, 1.14–1.50) during the first 15 years following T1DM onset, and the HR reached 1.01 (95% CI 0.97–1.05) after 15 years of follow-up. This pattern could be due to a greater earlier risk among people who are genetically susceptible, as only a subset of patients with T1DM will develop ESRD. In addition, the risk plateau associated with longer durations of T1DM may reflect an increased number of all-cause deaths in older T1DM patients.^31^ A lower risk of ESRD in older T1DM patients was also noted in our study; this may be attributed to other causes of death (eg, cardiovascular disease or cancer) or to a better prognosis in those T1DM long-term survivors.

The strengths of our study included its nationwide population-based cohort design, relatively long duration of follow-up (up to 12 years), and the large number of incident cases of T1DM (>7000); this allowed us to calculate the incidence and mortality of ESRD according to various sex and age stratifications. In addition, the study used the registration system of catastrophic diseases in Taiwan, which provides disease diagnosis validity. In addition, based on the universal coverage system, all patients with T1DM were enrolled in the study and a nationally representative sample can be assumed in our study. Findings from our study may provide up-to-date information on ESRD incidence of T1DM in Asian populations. Despite the above strengths, the limitations of our study must be considered. First, the claim data do not cover laboratory data and clinical details specific to T1DM, which make it difficult to determine the severity of T1DM and predicting factor of ESRD. Second, there is no information regarding the actual underlying cause of ESRD in the NHI claims; thus, we were unable to estimate the cause-specific risk of ESRD in T1DM. Third, we only included patients with a CIC for T1DM; some patients with T1DM who did not apply for a CIC for this condition were not included in the cohort. Finally, our study was based on a prevalence cohort instead of an incidence cohort and the patients were not followed up from the time of diagnosis; therefore, the cumulative risk of ESRD after diagnosis of T1DM could not be accurately estimated.

## CONCLUSIONS

We found that patients with T1DM were at a substantially increased risk of ESRD when compared with the general population in Taiwan. This ethnic information regarding the risk of progression to ESRD will help drive ongoing and future research efforts. Although there is continuously improved renal outcomes over time, the slightly higher SIR in female patients, especially the young group, should lead to more aggressive risk factor management in order to avoid the onset of ESRD in these high-risk patient populations of Taiwan.

## References

[1] Asao K Sarti C Forsen T Long-term mortality in nationwide cohorts of childhood-onset type 1 diabetes in Japan and Finland. *Diabetes Care* 2003; 26:2037–2042. 12832309 10.2337/diacare.26.7.2037 PMC3752687

[2] Nishimura R LaPorte RE Dorman JS Mortality trends in type 1 diabetes. The Allegheny County (Pennsylvania) Registry 1965–1999. *Diabetes care* 2001; 24:823–827. 11347737 10.2337/diacare.24.5.823

[3] Shankar A Klein R Klein BE Moss SE Association between glycosylated hemoglobin level and cardiovascular and all-cause mortality in type 1 diabetes. *Am J Epidemiol* 2007; 166:393–402. 17526864 10.1093/aje/kwm096

[4] Krolewski AS Warram JH Christlieb AR The changing natural history of nephropathy in type I diabetes. *Am J Med* 1985; 78:785–794. 3993659 10.1016/0002-9343(85)90284-0

[5] Cowie CC Port FK Wolfe RA Disparities in incidence of diabetic end-stage renal disease according to race and type of diabetes. *N Engl J Med* 1989; 321:1074–1079. 2797067 10.1056/NEJM198910193211603

[6] Stephens GW Gillaspy JA Clyne D Racial differences in the incidence of end-stage renal disease in types I and II diabetes mellitus. *Am J Kidney Dis* 1990; 15:562–567. 2368696 10.1016/s0272-6386(12)80527-0

[7] Lin WH Wang MC Wang WM Incidence of and mortality from type i diabetes in Taiwan from 1999 through 2010: a nationwide cohort study. *PLoS ONE* 2014; 9:e86172. 24465941 10.1371/journal.pone.0086172 PMC3899133

[8] Patterson CC Gyurus E Rosenbauer J Trends in childhood type 1 diabetes incidence in Europe during 1989–2008: evidence of non-uniformity over time in rates of increase. *Diabetologia* 2012; 55:2142–2147. 22638547 10.1007/s00125-012-2571-8

[9] U.S. Renal Data System (2012) Annual Data Report: Atlas of Chronic Kidney Disease and End-Stage Renal Disease in the United States: National Institutes of Health, National Institute of Diabetes and Digestive and Kidney Diseases, Bethesda, MD.

[10] Wen CP Cheng TY Tsai MK All-cause mortality attributable to chronic kidney disease: a prospective cohort study based on 462 293 adults in Taiwan. *Lancet* 2008; 371:2173–2182. 18586172 10.1016/S0140-6736(08)60952-6

[11] Matsushima M Tajima N LaPorte RE Markedly increased renal disease mortality and incidence of renal replacement therapy among IDDM patients in Japan in contrast to Allegheny County, Pennsylvania, USA. Diabetes Epidemiology Research International (DERI) U.S.-Japan Mortality Study Group. *Diabetologia* 1995; 38:236–243. 7713320 10.1007/BF00400100

[12] Krolewski M Eggers PW Warram JH Magnitude of end-stage renal disease in IDDM: a 35 year follow-up study. *Kidney Int* 1996; 50:2041–2046. 8943488 10.1038/ki.1996.527

[13] Xue JL Eggers PW Agodoa LY Longitudinal study of racial and ethnic differences in developing end-stage renal disease among aged medicare beneficiaries. *J Am Soc Nephrol* 2007; 18:1299–1306. 17329578 10.1681/ASN.2006050524

[14] Sun Y Chang YH Chen HF Risk of Parkinson disease onset in patients with diabetes: a 9-year population-based cohort study with age and sex stratifications. *Diabetes Care* 2012; 35:1047–1049. 22432112 10.2337/dc11-1511 PMC3329814

[15] Lin WH Guo CY Wang WM Incidence of progression from newly diagnosed systemic lupus erythematosus to end stage renal disease and all-cause mortality: a nationwide cohort study in Taiwan. *Int J Rheum Dis* 2013; 16:747–753. 24382283 10.1111/1756-185X.12208

[16] Lawrence JM Black MH Zhang JL Validation of pediatric diabetes case identification approaches for diagnosed cases by using information in the electronic health records of a large integrated managed health care organization. *Am J Epidemiol* 2014; 179:27–38. 24100956 10.1093/aje/kwt230

[17] Fine JP Gray RJ A proportional hazards model for the subdistribution of competing risk. *J Am Stat Assoc* 1999; 94:496–509.

[18] Finnish Registry for Kidney Diseases. Report 2012. Helsinki, Finland: Finnish Registry for Kidney Diseases; 2012 Available at: http://www.musili.fi/smtr/english.

[19] Finne P Reunanen A Stenman S Incidence of end-stage renal disease in patients with type 1 diabetes. *JAMA* 2005; 294:1782–1787. 16219881 10.1001/jama.294.14.1782

[20] Chen YR Yang Y Wang SC Effectiveness of multidisciplinary care for chronic kidney disease in Taiwan: a 3-year prospective cohort study. *Nephrol Dial Transplant* 2013; 28:671–682. 23223224 10.1093/ndt/gfs469

[21] Rosolowsky ET Skupien J Smiles AM Risk for ESRD in type 1 diabetes remains high despite renoprotection. *J Am Soc Nephrol* 2011; 22:545–553. 21355053 10.1681/ASN.2010040354 PMC3060448

[22] Costacou T Fried L Ellis D Orchard TJ Sex differences in the development of kidney disease in individuals with type 1 diabetes mellitus: a contemporary analysis. *Am J Kidney Dis* 2011; 58:565–573. 21840097 10.1053/j.ajkd.2011.05.025 PMC3183221

[23] Peralta CA Shlipak MG Fan D Risks for end-stage renal disease, cardiovascular events and death in Hispanic versus non-Hispanic white adults with chronic kidney disease. *J Am Soc Nephrol* 2006; 17:2892–2899. 16959827 10.1681/ASN.2005101122

[24] Hsu CY Lin F Vittinghoff E Shlipak MG Racial differences in the progression from chronic renal insufficiency to end-stage renal disease in the United States. *J Am Soc Nephrol* 2003; 14:2902–2907. 14569100 10.1097/01.asn.0000091586.46532.b4

[25] Brancati FL Whittle JC Whelton PK The excess incidence of diabetic end-stage renal disease among blacks. A population-based study of potential explanatory factors. *JAMA* 1992; 268:3079–3084. 1433738

[26] Hall YN Hsu CY Iribarren C The conundrum of increased burden of end-stage renal disease in Asians. *Kidney Int* 2005; 68:2310–2316. 16221234 10.1111/j.1523-1755.2005.00691.x

[27] Barbour SJ Er L Djurdjev O Differences in progression of CKD and mortality amongst Caucasian, Oriental Asian and South Asian CKD patients. *Nephrol Dial Transplant* 2010; 25:3663–3672. 20368302 10.1093/ndt/gfq189

[28] Karter AJ Ferrara A Liu JY Ethnic disparities in diabetic complications in an insured population. *JAMA* 2002; 287:2519–2527. 12020332 10.1001/jama.287.19.2519

[29] Chandie Shaw PK Baboe F van Es LA South-Asian type 2 diabetic patients have higher incidence and faster progression of renal disease compared with Dutch-European diabetic patients. *Diabetes Care* 2006; 29:1383–1385. 16732026 10.2337/dc06-0003

[30] Forsblom C Harjutsalo V Thorn LM Competing-risk analysis of ESRD and death among patients with type 1 diabetes and macroalbuminuria. *J Am Soc Nephrol* 2011; 22:537–544. 21335512 10.1681/ASN.2010020194 PMC3060447

[31] Lecaire TJ Klein BE Howard KP Risk for end-stage renal disease over 25 years in the population-based WESDR cohort. *Diabetes Care* 2014; 37:381–388. 24026564 10.2337/dc13-1287 PMC3898749

